# Taxonomy-aware, disorder-matched benchmarking of phase-separating protein predictors

**DOI:** 10.1186/s13059-026-04164-1

**Published:** 2026-06-17

**Authors:** Shuang Hou, Hexin Shen, Yong Zhang

**Affiliations:** State Key Laboratory of Cardiovascular Diseases and Medical Innovation Center, Institute for Regenerative Medicine, Department of Neurosurgery, Shanghai East Hospital, Shanghai Key Laboratory of Signaling and Disease Research, Frontier Science Center for Stem Cell Research, School of Life Sciences and Technology, Tongji University, Shanghai, 200092 China

**Keywords:** Phase-separating proteins, PSP predictors, Benchmarking, Taxonomy-aware evaluation

## Abstract

**Background:**

Biomolecular condensates formed via liquid–liquid phase separation (LLPS) play vital roles in cellular organization and function. Computational prediction of phase-separating proteins (PSPs) is increasingly used to prioritize candidates at proteome scale, making robust, well-designed benchmarks essential for fair evaluation and iterative improvement of PSP predictors.

**Results:**

We first show that a recently released PSP benchmark is substantially confounded by the imbalances in taxonomic origin and intrinsic-disorder compositions between positive and negative sets, allowing predictors to achieve high apparent performance by exploiting non-LLPS shortcuts and obscuring their true ability to distinguish PSPs. To minimize these effects, we construct a taxonomy-aware, disorder-matched PSP benchmark. Using this benchmark, we find that absolute sequence and biophysical feature values of PSPs differ markedly across taxa, whereas LLPS-associated feature shifts relative to taxon-specific proteome backgrounds are comparatively conserved. Benchmarking nineteen PSP predictors under this framework reveals pronounced taxon-dependent variation in performance. Moreover, PSPs lacking intrinsically disordered regions consistently constitute a more challenging regime across methods, motivating routine disorder-stratified evaluation.

**Conclusions:**

Our taxonomy-aware, disorder-matched benchmarking framework reduces shortcut-driven biases, enables more interpretable evaluation of PSP predictors, and provides guidance for developing models that capture transferable LLPS-associated signals rather than dataset- or taxon-specific shortcuts.

**Supplementary Information:**

The online version contains supplementary material available at 10.1186/s13059-026-04164-1.

## Background

Biomolecular condensates are widespread membraneless cellular compartments that spatially and temporally organize biochemical reactions and have emerged as a pervasive principle of intracellular organization [1, 2]. They support diverse biological functions ranging from transcriptional regulation to stress responses, and their dysregulation has been implicated in numerous diseases [3, 4]. Condensates often form through liquid–liquid phase separation (LLPS), a demixing process in which macromolecules spontaneously concentrate into a dense phase while remaining dynamic and reversible. Proteins that participate in LLPS are commonly referred to as phase-separating proteins (PSPs). Given the diversity of PSP roles and the limited scalability of experimental characterization, a wide range of computational PSP predictors has been developed to infer LLPS propensity [5–24]. Accurate prediction is valuable for proteome-wide prioritization of candidate PSPs, but the credibility of these predictors depends critically on the datasets used for training and evaluation. Multiple LLPS-focused databases annotate proteins observed in biomolecular condensates, yet they differ substantially in conceptual scope and inclusion criteria, leading to highly divergent annotations and protein coverage. For instance, LLPSDB is oriented toward in vitro experimentally validated LLPS systems [25]; PhaSePro is restricted to experimentally validated driver proteins or driver regions [26]; PhaSepDB adopts broader inclusion by distinguishing PS-self proteins that phase separate without partners from PS-other proteins that require partners such as proteins or RNA [27]; DrLLPS organizes LLPS-associated proteins by functional roles as scaffolds, clients, or regulators [28]. These conceptual differences make benchmark construction non-trivial and underscore why carefully designed benchmarks are essential for reproducible and fair comparisons across predictors. Recently, the benchmark introduced by Pintado-Grima and colleagues [29] represented an important step toward standardizing such comparisons by integrating curated positive and negative sets for systematic evaluation.

A key, often underappreciated complication is that the molecular grammars that encode condensate formation can vary across taxa. For example, yeast prions often share portable, intrinsically disordered prion domains enriched in asparagine, glutamine, tyrosine and glycine, whereas this domain architecture is not found in the mammalian prion protein or in the fungal prion HET-s and is rare in prokaryotes [30]. Such taxon dependence matters for benchmarking. As we show below, the Pintado-Grima benchmark couples PSP labels with taxonomic identity (with positives heavily enriched in mammals and negatives enriched in prokaryotes; see Results for details), and predictor performance on this benchmark is systematically associated with the similarity between a model’s training-taxonomic distribution and the benchmark’s composition. Under these conditions, benchmarks can reward taxon discrimination rather than LLPS-relevant biophysics, conflating genuine PSP generalization with differences in taxonomic composition.

A second axis of potential confounding arises from intrinsic disorder. Intrinsically disordered regions (IDRs) often contribute to multivalent interactions and are frequently associated with condensates, yet they are not universally necessary or sufficient for LLPS [31]. In parallel, LLPS can be driven by folded modular interaction domains that support multivalency through specific binding networks [31]. Therefore, a benchmark that inadvertently contrasts disorder-rich positives against disorder-poor negatives risks rewarding predictors that primarily detect disorder enrichment rather than LLPS-associated molecular grammars. This motivates controlling not only taxonomic composition, but also the balance of proteins with IDRs (IDPs) versus proteins without IDRs (non-IDPs) when constructing evaluation sets, in order to reduce shortcut learning.

Here, we address these issues by constructing a taxonomy-aware, disorder-matched PSP benchmark dataset designed to minimize the influence of taxonomic and disorder-related confounders. By balancing positives and negatives within major taxonomic groups and matching disorder composition between classes, our benchmark aims to prevent strong separation driven by taxonomic imbalance or disorder composition differences, thereby forcing evaluation to reflect LLPS-associated molecular grammars rather than trivial shortcuts. Using this benchmark, we show that absolute sequence and biophysical feature values of PSPs differ markedly across taxa, highlighting the importance of taxon-specific baselines. We therefore report PSP predictor performances separately for each major taxonomic group and further stratify by disorder status. Together, this framework enables more interpretable and trustworthy comparisons among PSP predictors and offers practical guidance for future model development.

## Results

### Taxonomic and disorder-composition biases in benchmark and training datasets

**Fig. 1:**
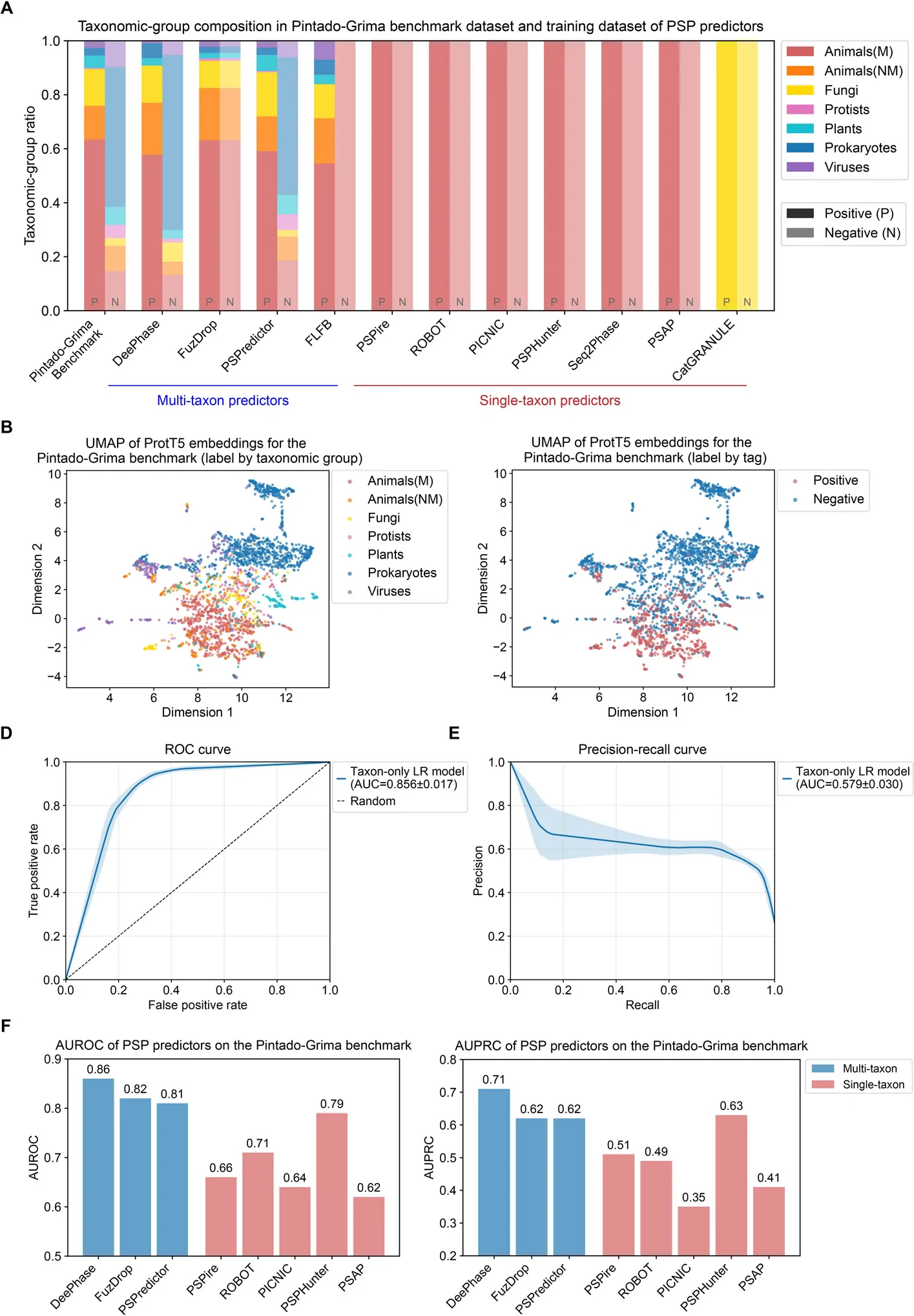
Taxonomic and disorder-composition biases confound PSP predictor benchmarking. **A** Taxonomic-group distributions of positives (P) and negatives (N) in the Pintado-Grima benchmark and in the reported training datasets of phase-separation protein (PSP) predictors. Stacked bars show the fraction of proteins assigned to each major taxonomic group: Animals(M), Animals(NM), Fungi, Protists, Plants, Prokaryotes, and Viruses. Animals(M) denotes mammals, and Animals(NM) denotes non-mammalian animals. Distribution profiles were computed separately for positive and negative sets within each dataset. Predictors are grouped by the taxonomic scope of their training data (as indicated below): multi-taxon predictors (trained on multiple taxa) and single-taxon predictors (trained on a single taxon). **B** and **C** UMAP projection of ProtT5 sequence embeddings for proteins in the Pintado-Grima benchmark, colored by taxonomic group (**B**) and by class label (positive versus negative; **C**). **D** and **E** Performance of a logistic regression classifier trained to predict Pintado-Grima benchmark labels using only one-hot encoded taxonomic-group composition as input features. Receiver operating characteristic (ROC; **D**) and precision-recall (**E**) curves are shown. Shaded regions indicate variability across dataset splits generated with different random seeds, and insets report mean area under the curve (AUC) ± s.d. **F** Bar plots of AUROC and AUPRC for PSP predictors evaluated on the Pintado-Grima benchmark dataset. For each predictor, AUROC (left) and AUPRC (right) were obtained directly from the Pintado-Grima benchmark article. Predictors are shown only when AUROC or AUPRC values were reported in the Pintado-Grima benchmark. These values were not available for some methods whose native outputs were not treated as explicit probabilistic scores in that benchmark. Predictors are grouped as multi-taxon predictors (blue bars) or single-taxon predictors (red bars)

To determine whether evaluations on the PSP benchmark introduced by Pintado-Grima and colleagues are confounded by taxonomic composition, we first quantified the taxonomic-group distributions of its positive and negative sets. We partitioned proteins into seven major taxonomic groups: Animals(M) (mammals), Animals(NM) (non-mammalian animals), Fungi, Protists, Plants, Prokaryotes, and Viruses. This analysis revealed a pronounced imbalance between labels: positives were dominated by mammals (63.4%), whereas negatives were dominated by prokaryotes (51.7%), resulting in substantially different taxonomic profiles between the two classes (Fig. 1A). Thus, taxonomic origin is strongly coupled to the benchmark labels, raising the possibility that performance estimates on this benchmark can be inflated by taxon-associated signals rather than PSP-specific determinants.

To examine whether the taxonomic structure is reflected in sequence representation space, we calculated ProtT5 [32] embeddings of proteins in benchmark dataset and visualized them using UMAP [33]. Benchmark proteins segregated strongly by taxonomic group in the embedding space (Fig. 1B), indicating that taxon-linked sequence features dominate the low-dimensional geometry. Notably, the apparent separation between positives and negatives largely mirrored this taxon-driven organization (Fig. 1C), suggesting that the observed label separation can be largely explained by taxonomic structure.

If taxonomy indeed encodes label-associated information in this benchmark, then a classifier that uses only taxonomic identity should achieve non-trivial performance. We therefore trained a logistic regression model with one-hot encoded taxonomic-group indicators as the sole input features. Despite the complete absence of sequence-related features, this taxonomy-only baseline achieved measurable discrimination (mean AUROC = 0.856 ± 0.017; mean AUPRC = 0.579 ± 0.030 across random splits; Fig. 1D). These results provide direct evidence that taxonomic identity itself contains substantial label information in the Pintado-Grima benchmark, establishing taxonomy as a concrete confounder for predictor evaluation.

One practical consequence of this label-taxonomy coupling is that performance estimates may become sensitive to how closely a predictor’s training data match the benchmark’s taxonomic composition. Predictors could be broadly classified into two groups based on the taxonomic composition of their training datasets: multi-taxon predictors trained on multiple taxa, and single-taxon predictors trained on a single taxon (Animals(M)). Notably, among the four multi-taxon predictors, DeePhase, PSPredictor, and FLFB exhibited marked differences in taxonomic composition between their positive and negative training sets, resembling the label-associated taxonomic imbalance observed in the Pintado-Grima benchmark. In addition, the positive training sets of the four multi-taxon predictors showed taxonomic distributions closely resembling that of the Pintado-Grima benchmark positive set (Fig. 1A). Accordingly, multi-taxon predictors generally outperformed single-taxon predictors (Fig. 1F), consistent with their closer taxonomic match to the benchmark dataset. Together with the taxon-structured embedding geometry (Fig. 1B) and the non-trivial performance of a taxonomy-only baseline (Fig. 1D), these results indicate that evaluation on the Pintado-Grima benchmark is intrinsically confounded by taxonomy, such that reported performance cannot be interpreted as a clean measure of PSP-discriminative ability.

Beyond taxonomic composition, predictors vary widely in training dataset size (Additional file 1: Fig. S1A) and differ systematically in disorder composition (Additional file 1: Fig. S1B, C), which can shape benchmark performance. In particular, the negative sets used to train multi-taxon predictors show IDR-proportion distributions that are closer to the Pintado-Grima benchmark negatives, whereas negatives from single-taxon predictors tend to exhibit higher IDR proportions (Additional file 1: Fig. S1B). Likewise, the fraction of non-IDPs among multi-taxon negatives more closely matches the Pintado-Grima benchmark negative set (both exceeding 50%), while the corresponding positives remain overwhelmingly IDP-rich (non-IDP fraction < 15%; Additional file 1: Fig. S1C). By contrast, single-taxon predictors show a less pronounced disparity in IDP/non-IDP fraction between their positive and negative training sets (Additional file 1: Fig. S1C). These patterns suggest that the apparent advantage of multi-taxon predictors on the Pintado-Grima benchmark may reflect not only taxonomic composition matching (Fig. 1F) but also closer matching of disorder composition, particularly within the negative set, to the benchmark. Together with the label-dependent taxonomic imbalance in the benchmark (Fig. 1A), these observations underscore the need for a taxonomy-aware benchmark that balances positives and negatives within each taxonomic group and matches the IDP/non-IDP ratio between labels, thereby forcing predictors to rely on LLPS-relevant features rather than taxonomy- or disorder-associated shortcuts.

We finally quantified overlap between predictor’s training sets and the Pintado-Grima benchmark dataset using the Jaccard index, separately for the positive and negative sets. Since the predictors were evaluated on the Pintado-Grima dataset without excluding their training sets, this analysis provides a direct estimate of potential data leakage. As shown in Additional file 1: Fig. S1D, the four multi-taxon predictors generally exhibited greater overlap with the Pintado-Grima dataset than the single-taxon predictors. This pattern was most pronounced for PSPredictor, which showed the highest overlap for both the positive and negative sets. By contrast, the single-taxon predictors showed only modest overlap for positives and near-zero overlap for negatives. Notably, the two multi-taxon predictors with available negative training sets, DeePhase and PSPredictor, displayed substantially higher overlap with the Pintado-Grima dataset than the single-taxon predictors. These results indicate that overlap with the Pintado-Grima benchmark is highly unequal across predictors and may contribute to data leakage, suggesting that part of the apparent performance advantage on this benchmark could reflect differential training-benchmark overlap rather than genuine generalization.

### Construction of a taxonomy-aware, disorder-matched PSP benchmark dataset

**Fig. 2:**
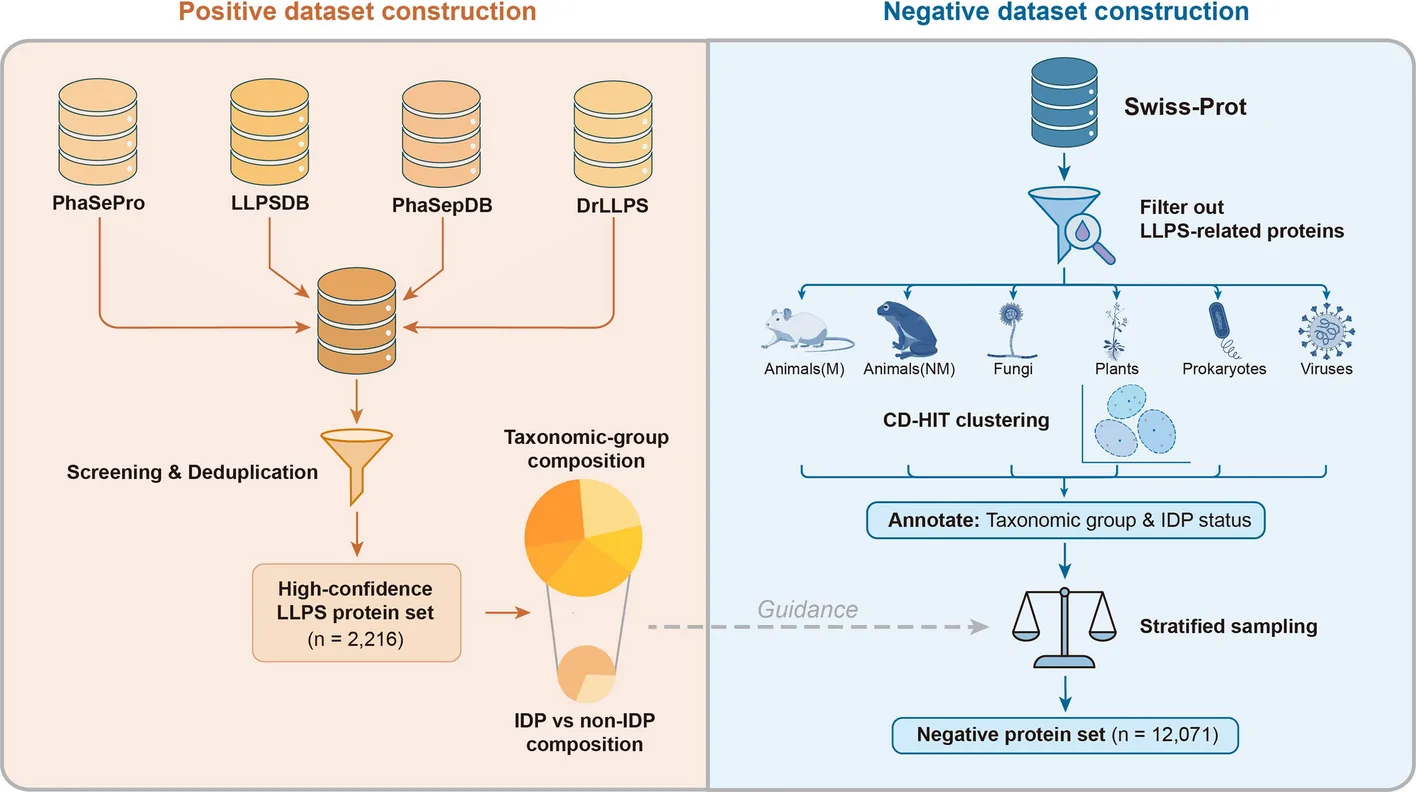
Taxonomy-aware construction of positive and negative datasets for PSP benchmarking. For the positive dataset, candidate proteins were compiled from four curated LLPS-focused resources (PhaSePro, LLPSDB, PhaSepDB and DrLLPS), merged, screened, and deduplicated to generate a high-confidence LLPS protein set (*n* = 2,216). The resulting taxonomic-group composition and the proportion of IDPs versus non-IDPs were summarized and used to guide negative-set sampling. For the negative dataset, candidate proteins were drawn from Swiss-Prot across major taxonomic groups (Animals(M), Animals(NM), Fungi, Plants, Prokaryotes and Viruses). Proteins with known or putative LLPS association were first filtered out, including the compiled PSPs, proteins showing sequence similarity to PSPs, known interactors of positive proteins, and proteins used as positives in the available training sets of evaluated predictors. Redundancy was then reduced by CD-HIT clustering within each taxonomic group. The remaining proteins were annotated by taxonomic group and IDP status and selected by stratified sampling to match the positive set’s taxonomic-group and IDP/non-IDP composition, yielding the final negative protein set (*n* = 12,071). Representative species icons were obtained from BioGDP [34]

Motivated by the strong coupling between PSP labels and taxonomic identity in the Pintado-Grima benchmark dataset and the IDP composition mismatches observed across training datasets, we constructed a new taxonomy-aware benchmark that explicitly controls two major confounders: (i) taxonomic composition and (ii) IDP/non-IDP composition (see Methods for details). Conceptually, the dataset was built such that (i) negatives are sampled to match the taxonomic-group composition of positives, and (ii) within each major taxonomic group, negatives are sampled to match the IDP/non-IDP composition of the corresponding positives (Additional file 2: Table S1). An overview of the construction workflow is shown in Fig. 2 and summarized as a stepwise Sankey diagram in Additional file 1: Fig. S2.

We first assembled the positive set by merging entries from four curated LLPS-focused databases (LLPSDB [25], PhaSePro [26], PhaSepDB [27], and DrLLPS [28]), followed by screening and de-duplication to obtain a high-confidence, non-redundant collection of 2,216 proteins spanning the major taxonomic groups (Animals(M), Animals(NM), Fungi, Plants, Prokaryotes, and Viruses; Fig. 2; Additional file 1: Fig. S2A). Protists were excluded due to insufficient positives. To characterize how different resources contribute to the final positive compendium, we summarized cross-database overlap using an UpSet plot [35] (Additional file 1: Fig. S3A), which highlights both shared cores and complementary coverage across resources. We futher quantified the distribution of the positive set across major taxonomic groups as well as its IDP versus non-IDP composition, and used these profiles as the target composition for taxonomy-aware negative sampling (Fig. 2; Additional file 1: Fig. S3B, C).

For the negative dataset, we compiled candidate proteins from Swiss-Prot across the same taxonomic groups as positives (Fig. 2; Additional file 1: Fig. S2B). Within each taxonomic group, we first performed redundancy reduction via CD-HIT clustering [36] to prevent over-representation of closely related sequences. To minimize contamination by true or putative PSPs (and to reduce information leakage through close homologs), we then removed proteins with known or suspected LLPS association. This filtering step included (i) the compiled PSP positives themselves and (ii) additional proteins exhibiting sequence similarity to PSPs. The resulting filtered pool was annotated by taxonomic group and IDP status, enabling stratified sampling to match the positive set’s composition. This procedure yielded a final negative set of 12,071 proteins. As expected, the resulting negative set closely matched the taxonomic-group composition of the positive set (Additional file 1: Fig. S3B, C). We further examined protein length distributions of the positive benchmark set, negative benchmark set, and the corresponding proteome background. As shown in Additional file 1: Fig. S3D, the negative set showed a length distribution similar to that of the proteome background, indicating that our negative set construction did not introduce an obvious length bias relative to the background pool. In contrast, the positive set showed a distinct length distribution compared with both the negative set and the proteome background. This difference is consistent with the known enrichment of long, multivalent, and often disordered proteins among curated PSPs, rather than being introduced by our negative sampling procedure. By design, this taxonomy-aware, disorder-matched benchmark minimizes taxonomy- and disorder-based shortcuts, making cross-taxon comparisons and taxonomy-stratified PSP benchmarking more interpretable.

To explicitly quantify sequence similarity within and between taxonomic groups, we computed pairwise global sequence identity for all protein pairs in the final positive and negative benchmark datasets. We used a global-alignment-based identity measure because it provides a stringent assessment of overall sequence similarity and avoids potential overestimation from short locally conserved motifs or domains (see Methods for details). We first visualized the resulting pairwise global identity values using heatmaps, with proteins grouped by taxonomic annotation (Additional file 1: Fig. S4A). Both the positive- and negative-set heatmaps showed broadly low pairwise global identities and no prominent high-identity blocks. We further summarized the distributions of pairwise global identity for each within- and between-taxon comparison using boxplots (Additional file 1: Fig. S4B). These analyses showed low sequence similarity across both within-taxon and between-taxon comparisons in the positive and negative datasets, with median pairwise global identities below 15% in both cases. These results indicate that the benchmark is largely free of high sequence redundancy and that the observed taxon-resolved patterns are unlikely to be explained by high sequence similarity within specific taxonomic groups.

### Taxon-specific baselines shape LLPS-associated sequence and biophysical features

**Fig. 3:**
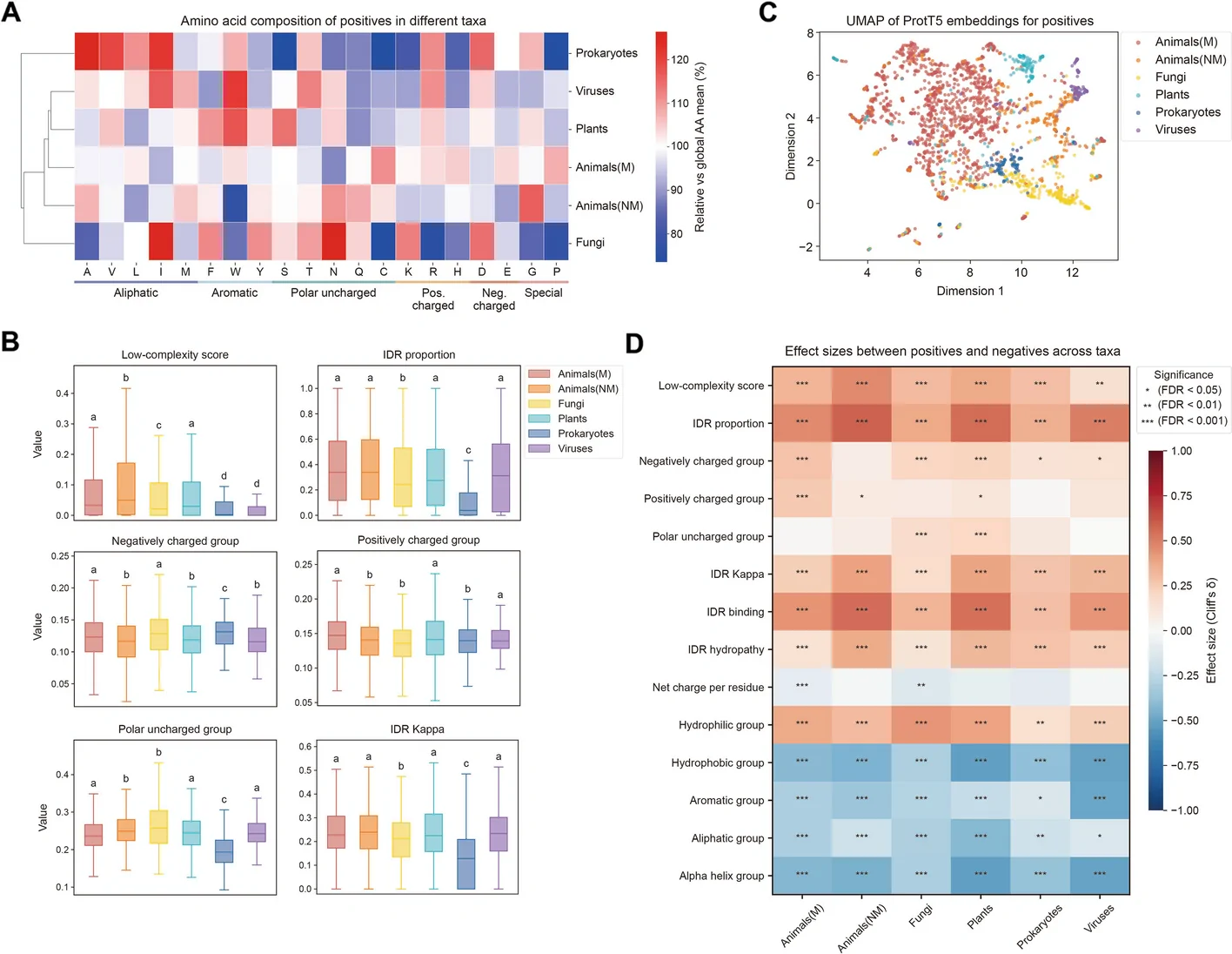
Taxon-dependent sequence and biophysical properties of PSPs reflect distinct proteome baselines. **A** Amino-acid composition of PSPs across taxonomic groups. Heatmap entries represent ${R}_{s,aa}$, defined as the ratio of the mean frequency of each amino acid in a given taxonomic group to its global mean frequency across all proteins. A value of 1 indicates the global mean, values greater than 1 indicate enrichment, and values less than 1 indicate depletion. Taxa were hierarchically clustered using Euclidean distance calculated from the ${R}_{s,aa}$ values and average linkage. Amino acids are grouped by physicochemical class (aliphatic, aromatic, polar uncharged, positively charged, negatively charged and special). **B** Distributions of representative sequence/biophysical features for positives across taxa, including low-complexity score, intrinsically disordered region (IDR) proportion, the fraction of negatively charged residues (D, E), the fraction of positively charged residues (K, R, H), the fraction of polar uncharged residues (S, T, N, Q, C), and IDR Kappa (κ; charge patterning). Box plots show median (central line), interquartile range (IQR; 25th-75th percentiles), and whiskers extending to the most extreme values within 1.5 × IQR. Letters denote statistical groupings from pairwise comparisons among taxonomic groups within each feature. For each feature, two-sided Mann–Whitney U tests were performed between taxonomic groups, followed by BH-FDR correction. Taxonomic groups assigned the same letter are not significantly different, whereas taxonomic groups assigned different letters differ significantly at BH-FDR < 0.05. **C** UMAP projection of ProtT5 embeddings for positive proteins, colored by taxonomic group. **D** Effect sizes between PSPs and proteome background across taxa. Heatmap shows the taxon-stratified effect size (Cliff’s δ) for representative sequence/biophysical features. Color intensity reflects the magnitude of the effect size. Asterisks denote statistical significance from two-sided Mann–Whitney U tests with BH-FDR correction; the number of asterisks indicates the FDR tier (*** indicates FDR < 0.001, ** indicates FDR < 0.01, and * indicates FDR < 0.05)

Using our taxonomy-aware, disorder-matched benchmark, we next examined how sequence and biophysical features associated with PSPs vary across taxa. To this end, we analyzed the PSPs in the positive set in a taxonomy-stratified framework. We first examined whether PSP sequence composition is conserved across taxa. For each amino acid, we computed the relative change in mean residue frequency within a given taxon compared with the global mean across all taxa (Fig. 3A). The resulting heatmap is far from uniform: each taxon exhibits a distinct signed pattern across residues, and many amino acids flip direction (enriched in one taxon but depleted in another). For example, Prokaryotes PSPs show a striking enrichment of aliphatic residues, with alanine, valine, leucine, and isoleucine all strongly enriched, whereas Fungi PSPs display a split aliphatic signature in which isoleucine is strongly enriched but alanine and valine are clearly depleted. Even within animals, Animals(NM) PSPs show striking depletion of tryptophan and enrichment of glycine, while Animals(M) PSPs display comparatively modest deviations. To improve visualization of taxon-level relationships in amino-acid composition, we hierarchically clustered taxonomic groups based on their relative residue-composition profiles, thereby highlighting similarities and differences in compositional patterns directly within the heatmap (Fig. 3A).

We then examined whether commonly used LLPS-associated properties show similar taxon dependence. We quantified a panel of disorder-, charge-, and composition-related descriptors, with Fig. 3B showing representative features (low-complexity score, IDR proportion, and the fractions of negatively charged, positively charged, polar uncharged residues, and IDR κ [37]), and Additional file 1: Fig. S5 extending the analysis to additional IDR-centric and compositional properties (IDR binding score [38, 39], IDR hydropathy [37], NCPR, and multiple residue-group fractions) (see Methods for details). The analysis revealed that feature distributions were broadly taxon dependent (Fig. 3B; Additional file 1: Fig. S5). A prominent example is the contrast between Prokaryotes and Animals(M): relative to Animals(M), Prokaryotes PSPs exhibit markedly lower IDR proportion and low-complexity scores, reduced IDR κ and IDR binding scores, together with strikingly higher hydrophobic and aliphatic fractions and an elevated α-helix-associated residue fraction. Consistent with these feature-level heterogeneity, a UMAP projection [33] of ProtT5 embeddings [32] of PSPs revealed clear taxon-linked organization (Fig. 3C).

Since many sequence properties vary across taxa at the proteome level, we further examined whether the heterogeneity observed above could be explained simply by taxon-specific proteome baselines. We therefore performed a within-taxon background-contrast analysis: for each taxonomic group, we compared PSPs against the corresponding taxon-specific proteome background and quantified PSP-background separation for each feature using Cliff’s δ [40] (Fig. 3D). While some feature-taxon pairs show weak or non-significant separation, for a majority of features, PSPs deviate from their own proteome baselines in a consistent direction (Fig. 3D). This indicates that much of the apparent cross-taxon heterogeneity in absolute feature values reflects taxon-specific proteome baselines, whereas LLPS-associated feature shifts relative to background are comparatively conserved, highlighting that absolute feature levels are not directly comparable across taxa.

To assess whether the full-set cross-taxon feature differences also occur among homologous PSPs, we further performed a homolog-restricted sensitivity analysis using OMA orthogroups. Although we initially sought multi-taxon shared orthogroups, sufficient sample sizes were obtained only for two-taxon paired comparisons, namely Animals(M)-Fungi and Animals(M)-Animals(NM) (see Methods for details; Additional file 1: Fig. S6). Almost all features were not significantly different between these two paired orthogroups, with only one feature reaching significance in the Animals(M)-Fungi comparison. Moreover, the paired effect sizes did not reveal a consistent directional pattern across features or taxon pairs. These findings indicate that broad or systematic feature divergence between taxa was not evident within the limited set of directly matched PSP orthogroups. This homolog-restricted analysis therefore refines the interpretation of full-set comparison by indicating that the observed feature heterogeneity is more likely to arise from differences in PSP repertoire composition, taxon-specific proteome baselines, and taxon-associated cellular or environmental conditions than from uniform physicochemical divergence among homologous PSPs.

Because full-set feature heterogeneity may reflect differences in the types of PSPs represented in each taxon, we next characterized the functional roles and domain compositions of PSPs within each taxonomic group. GO and Pfam enrichment analyses revealed taxon-specific enrichment patterns (Additional file 1: Fig. S7A, B), indicating that PSPs from different taxa are associated with distinct functional repertoires and domain-architecture contexts rather than representing a single compositionally uniform protein class. Together, these analyses indicate that the observed cross-taxon sequence and biophysical heterogeneity may reflect not only differences in the underlying taxon-specific proteome baselines, but also differences in the functional repertoires and domain architectures represented in each taxonomic group. These results support the need to interpret PSP properties relative to taxon-specific baselines and to evaluate PSP predictors in a taxon-resolved rather than pooled multi-taxon setting.

### Taxonomic- and disorder-dependent performance of PSP predictors

Using our taxonomy-aware, disorder-matched benchmark, we compared PSP predictors in six taxonomic groups: Animals(M), Animals(NM), Fungi, Plants, Prokaryotes, and Viruses. Nineteen predictors available before January 2026 (see Methods for details) were evaluated and grouped into two main categories (Additional file 3: Table S2): (i) rule-based, heuristic models (PLAAC [5], PScore [7], and PSPer [8]); and (ii) machine-learning (ML)-based supervised models. The supervised models were further subdivided by input representation into engineered/physicochemical feature-based models (FuzDrop [9], PSAP [12], PhaSePred [13], PICNIC [15], PSPire [16], FLFB [17], and ROBOT [24]), protein language model (PLM) embedding-based models (Seq2Phase [18], PSTP [20], PhaseNet [21], MambaPhase [22], and PSPsPredict [23]), and other representation-learning models (Droppler [10], DeePhase [11], PSPredictor [14], and PSPHunter [19]). Predictor performance across taxonomic groups was quantified using both threshold-free metrics (AUROC, AUPRC, and AUPRC ratio) and threshold-dependent metrics (MCC, F1-score, precision, recall, specificity, accuracy, and positive likelihood ratio). To avoid the influence of class imbalance, predictor evaluation was performed on balanced evaluation sets generated by repeated negative subsampling (see Methods for details). The mean and variance of the evaluation metrics are reported in Additional file 4: Table S3, and the distributions of AUROC and AUPRC values across repeats on the Animals(M) subset are shown as examples in Additional file 1: Fig. S8.

**Fig. 4:**
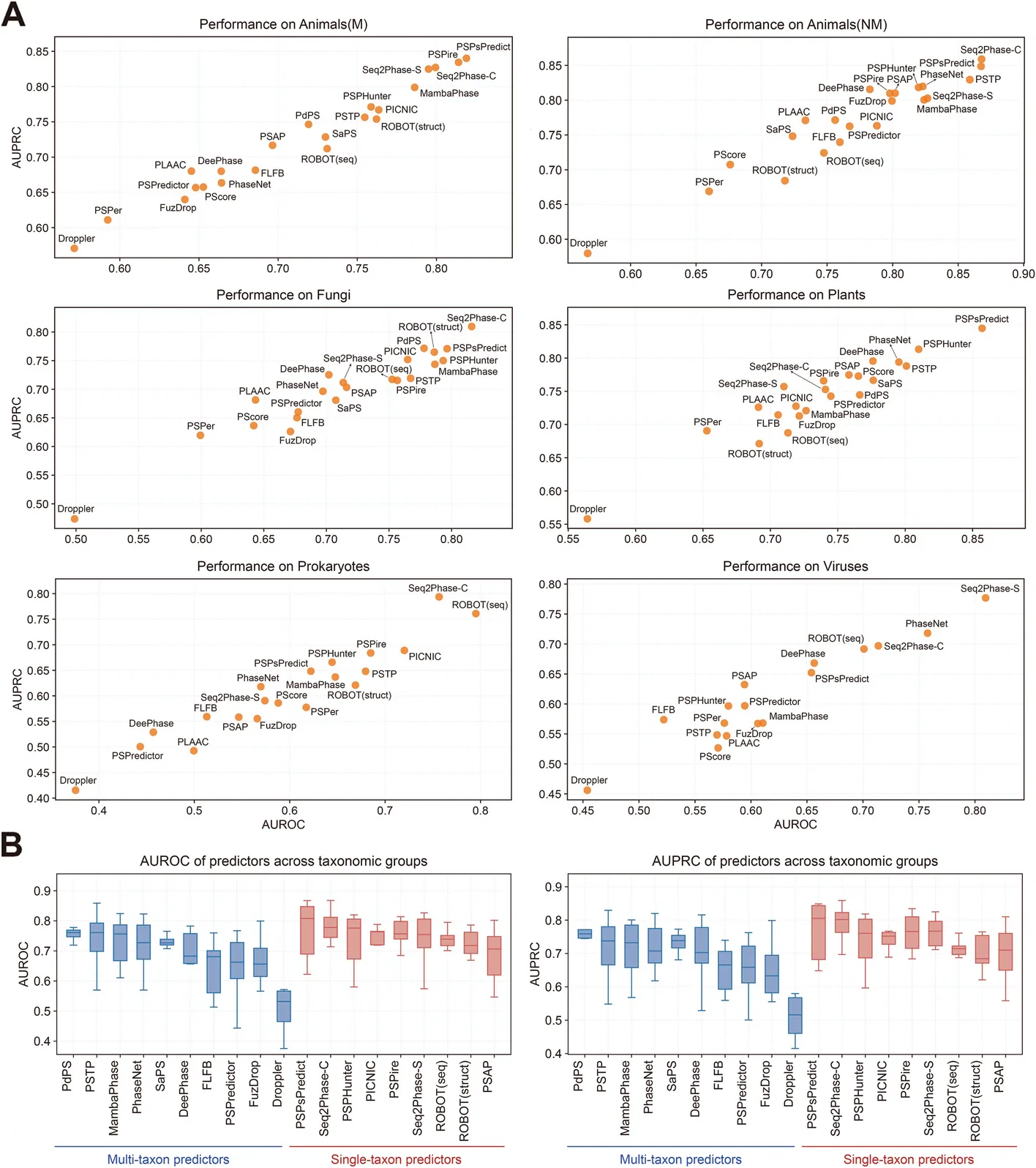
Performance of PSP predictors on the benchmark dataset. The training positive sets of all evaluated predictors were removed from the benchmark dataset before evaluation, except for Droppler, whose training positive set was unavailable. **A** Scatter plots show mean AUROC versus AUPRC across ten negative subsampling repeats, with each point labelled by predictor name. The two PhaSePred variants (SaPS for self-assembling proteins and PdPS for partner-dependent proteins) and the two Seq2Phase variants (Seq2Phase-C targeting client proteins and Seq2Phase-S targeting scaffold proteins) were evaluated separately. ROBOT was also evaluated separately using its two supported input types: ROBOT(seq), based on protein sequence input, and ROBOT(struct), based on structural (PDB) input. Performance was evaluated separately across six taxonomic groups: Animals(M), Animals(NM), Fungi, Plants, Prokaryotes, and Viruses. The two PhaSePred variants (SaPS and PdPS) were excluded from evaluation on the Prokaryotes subset due to limited protein support; while PhaSePred, PSPire, ROBOT(struct), and PICNIC were excluded from evaluation on the Viruses subset for the same reason. **B** Distribution of AUROC and AUPRC values of predictors across taxonomic groups. Predictors are grouped by the taxonomic scope of their training data (as indicated below): multi-taxon predictors (trained on multiple taxa) and single-taxon predictors (trained on a single taxon). Since the three rule-based, heuristic models (PLAAC, PScore, and PSPer) do not require training data, they were not shown. Boxplots show median (central line), interquartile range (IQR; 25th-75th percentiles), and whiskers extending to the most extreme values within 1.5 × IQR

Across these six taxonomic groups, both AUROC- and AUPRC-based rankings varied markedly (Fig. 4A). Unless otherwise stated, we refer to AUROC-based ranking in the text, but AUPRC showed broadly similar patterns. Rule-based, heuristic predictors generally show lower performance than ML-based supervised models across most taxa, suggesting that fixed heuristics capture only a limited subset of LLPS-relevant signals. Among supervised predictors, PLM embedding-based models (Seq2Phase, PSTP, PhaseNet, MambaPhase and PSPsPredict) typically achieved the strongest performance, frequently ranking among the top five across multiple taxa, indicating that PLM-derived embeddings provide rich sequence and biophysical representations that benefit PSP prediction. Several engineered/physicochemical feature-based models performed well in specific taxonomic groups, with PICNIC, PSPire, and ROBOT ranking among the top five in one, two, and three taxa, respectively. The other representation-learning models DeePhase and PSPHunter also achieved top-five performance in one or two taxa. These taxon-resolved results underscore that a single aggregate score on a pooled multi-taxon dataset (pooled evaluation, see Additional file 1: Fig. S9A) can obscure substantial differences in behavior across taxa and may be dominated by mixture effects rather than stable generalization across taxonomic groups. Other metrics showed similar patterns (Additional file 4: Table S3).

To further assess whether predictor performance was influenced by sequence similarity between predictor training positives and benchmark positives, we performed a stringent training-similarity filtering analysis (see Methods for details). Because too few positives remained for Animals(NM), Prokaryotes, and Viruses after filtering, the evaluation was restricted to Animals(M), Fungi, and Plants (Additional file 1: Fig. S9B-D). To assess whether this stricter filtering altered the comparative conclusions, we calculated Kendall’s tau correlation coefficient between predictor AUROC rankings before and after filtering for each retained taxonomic group. Ranking consistency remained high across all three taxonomic groups (Kendall’s tau = 0.775 for Animals(M), 0.922 for Fungi, and 0.974 for Plants), indicating that the additional 30% training-similarity filtering did not substantially alter the overall predictor rankings. Other metrics showed similar patterns (Additional file 5: Table S4).

On our taxonomy-aware, disorder-matched benchmark, the dependence of predictor performance on training-benchmark taxonomic similarity observed in the Pintado-Grima benchmark (Fig. 1F) was absent. Multi-taxon and single-taxon predictors exhibited broadly overlapping AUROC and AUPRC distributions across taxonomic groups (Fig. 4B). These results indicated that evaluation based on our benchmark dataset is no longer driven by simple taxonomic matching between training data and benchmark composition.

Our previous study showed that PSP predictors can behave differently on PSPs with IDRs (ID-PSPs) versus those without IDRs (noID-PSPs) [16]. Therefore, we stratified positives by disorder status to quantify performance gaps between these two classes. We evaluated ID-PSP and noID-PSP performance on the Animals(M) subset, as the remaining taxonomic groups contained too few noID-PSPs to support reliable estimation. Across all predictors tested, identifying noID-PSPs was consistently more challenging than identifying ID-PSPs, yielding a uniform decrease in AUROC (Additional file 1: Fig. S10). The magnitude of this performance gap varied substantially across predictors, indicating that current predictors differ widely in their ability to handle noID-PSPs. PSPire, MambaPhase, and Seq2Phase achieved the highest performance on noID-PSPs, whereas several predictors showed limited discrimination in this setting. Together, these results indicate that noID-PSPs represent a distinct and more challenging regime for current predictors, motivating routine disorder-stratified reporting for fair interpretation.

## Discussion

In computational biology, benchmarks are often treated as neutral arbiters of method quality. The recently released Pintado-Grima benchmark [29] has begun to play this role for PSP predictors. However, quantitative analysis of this dataset reveals systematic departures from such neutrality. We find that this mixed-taxon benchmark couples PSP labels to taxonomic identity and disorder composition, and that models can achieve high scores by exploiting benchmark-specific shortcuts rather than capturing transferable LLPS-associated grammars. Consistent with this, the Pintado-Grima benchmark can systematically favour predictors trained on multi-taxon datasets over single-taxon predictors, independent of their performance within their intended taxonomic group. To address this, our taxonomy-aware, disorder-matched benchmark is designed explicitly to minimize these effects, and reveals substantial taxon- and disorder-dependent variation in PSP predictor performance that pooled, shortcut-prone evaluations obscure. Importantly, our results should not be interpreted as evidence that taxon-specific PSP classifiers are necessarily required. Several predictors trained on single-taxon retain strong performance across multiple taxonomic groups.

A further consideration is that protein sequence variation alone may not fully explain the taxon-dependent differences in sequence and biophysical properties. LLPS is strongly influenced by cellular and environmental context, including ionic strength, pH, temperature, macromolecular crowding, and transcriptome composition, which can differ substantially across organisms and cellular states. These contextual factors may shape the apparent physicochemical requirements for LLPS. Thus, the observed cross-taxon differences in PSP properties may reflect not only taxon-specific proteome baselines and PSP repertoire composition, but also context-dependent cellular and environmental constraints. Incorporating such factors into future benchmarks may further improve the biological interpretability of PSP predictor evaluation.

Although our benchmark controls for taxonomic composition and IDP/non-IDP status, it does not explicitly match positives and negatives for all LLPS-relevant physicochemical and interaction-related properties, such as hydrophobicity, net charge, α-helix propensity, and RNA-binding propensity. These properties can influence condensate formation and may also affect predictor performance. We did not use these features as additional matching variables because they are continuous rather than categorical, mutually correlated, taxon dependent, and lack universally accepted thresholds for defining comparable LLPS-relevant regimes across taxa and PSP classes. Matching all of these properties simultaneously would require arbitrary discretization and high-dimensional stratified sampling, which would substantially restrict the available negative pool and potentially distort the taxonomic and disorder composition that our benchmark aims to control. Therefore, taxonomy and disorder status were retained as the primary benchmark-control variables, while incomplete control of other physicochemical regimes remains a limitation of the present study. Future benchmarks with larger and more uniformly annotated datasets could incorporate multi-property matching or model-based adjustment to further disentangle the effects of these properties.

In practice, studies that introduce a new predictor typically enforce that their independent test set is non-redundant with respect to their own training data, often considering both positive and negative proteins. However, when a benchmark aims to evaluate multiple previously published predictors, it is rarely feasible to guarantee simultaneous exclusion of all training sets. Training lists are frequently incomplete, inconsistently reported, or unavailable, making comprehensive decontamination impractical. In this work, to improve fairness while maintaining a realistic evaluation setting, we curated and removed the training positive sets of all predictors for which training data were obtainable; for methods where training positives were not publicly released, we attempted to obtain them by contacting the authors (see Methods for details). Droppler was the only predictor for which the training positive set remained unavailable despite our attempt to contact the authors; consequently, training-positive decontamination could not be performed for this method, and its benchmark performance should be interpreted with caution. In contrast, we did not remove training negatives, because the negative sets used by some predictors can be extremely large. An extreme case is PSAP [12], which treats the non-positive human proteome as negatives, yielding approximately 19,000 negative proteins. Removing such negatives would eliminate a substantial fraction of the candidate negative pool and could also alter the taxonomic and disorder composition that our benchmark was designed to control. To quantify the overlap between predictors’ training negative sets and the benchmark negative set, we computed the Jaccard index. All resulting Jaccard index values were below 0.1, indicating that overlap between our benchmark negatives and available negative training sets was limited (Additional file 1: Fig. S11). We therefore prioritized positive-set decontamination as the most direct and practical safeguard against performance inflation, while acknowledging remaining negative-set overlap as a limitation of multi-method benchmarking.

Our benchmark results also suggest practical directions for future PSP predictor design. The consistent underperformance of rule-based approaches implies that fixed heuristic rules capture only a limited subset of relevant biophysical signals. By contrast, predictors based on PLM embeddings exhibit comparatively robust performance across taxonomic groups, supporting the use of PLM-derived representations as a strong default backbone for PSP prediction. At the same time, the good performance of PSPire [16] on mammals highlights the added value of context-dependent regulatory features, such as post-translational modification annotations. Together, these observations motivate a hybrid architecture in which PLM embeddings provide the core representation for cross-taxon generalization, while lightweight modules incorporate regulatory and cellular-context features where appropriate.

## Conclusions

In this work, we show that benchmarking of PSP predictors can be strongly distorted by dataset structure, particularly when PSP labels are coupled to taxonomic origin and disorder composition. By constructing a taxonomy-aware, disorder-matched benchmark, we reduce these shortcut-driven effects and provide a more robust framework for evaluating PSP prediction methods across major taxonomic groups. Using this framework, we demonstrate that PSPs differ substantially in their absolute sequence and biophysical properties across taxa, whereas their deviations from taxon-specific proteome backgrounds are more conserved. These findings indicate that PSP properties and predictor performance should be interpreted relative to taxon-specific baselines rather than solely through pooled multi-taxon evaluations. Our systematic assessment of nineteen predictors further reveals pronounced taxon-dependent performance differences and identifies PSPs without IDRs as a consistently more challenging prediction regime. Together, these results establish taxonomy-aware and disorder-stratified benchmarking as an important requirement for fair PSP predictor evaluation, and provide practical guidance for developing future models that capture transferable LLPS-associated signals instead of dataset-, taxonomy-, or disorder-associated shortcuts.

## Methods

### Taxonomic composition analysis

To characterize taxonomic composition, we analyzed the benchmark dataset introduced by Pintado-Grima et al*.* [29] (2,876 proteins), together with the training datasets of predictors evaluated in that study. Training datasets were collected from original publications, supplementary materials, or publicly available repositories when available. As training datasets of Droppler [10] and FLFB [17] could not be obtained, we contacted the authors and received the FLFB training data which were provided as sequence fragments, but did not receive a response from the Droppler authors. Accordingly, Droppler was excluded from the taxonomic composition profiling. For FLFB, sequence fragments were mapped to full-length proteins by BLASTP [41] against Swiss-Prot. Fragments that were fully contained within a single protein and produced a unique match were assigned to that protein and used for downstream analyses. In addition, the four rule-based and heuristic models (PLAAC [5], PScore [7], PSPer [8], and R + Y [42]) were excluded because no explicit training datasets are defined for these methods. For PSPredictor [14], whose training data were also provided as sequence fragments, we mapped fragments to full-length proteins using the same BLASTP-based procedure applied to FLFB.

Taxonomic annotations were retrieved from UniProtKB and mapped to higher-level taxonomic lineages. Each protein was assigned to one of seven taxonomic groups: mammals (Animals(M)), non-mammalian animals (Animals(NM)), Fungi, Protists, Plants, Prokaryotes, or Viruses. Taxonomic distribution profiles were computed separately for the positive and negative sets within each dataset.

### Taxon-only classification model on the Pintado-Grima benchmark dataset

To quantify the predictive power of taxonomic information alone on the Pintado-Grima benchmark, we trained a classifier using only taxonomic group labels as input. Each protein was encoded as a one-hot vector indicating its assigned taxonomic group, and a logistic regression model was fit to predict class labels. Performance was evaluated by randomly splitting the benchmark dataset into training and test sets at an 8:2 ratio. This procedure was repeated for ten independent splits using different random seeds. Predictive performance was summarized using the area under the receiver operating characteristic curve (AUROC) and the area under the precision-recall curve (AUPRC).

### Intrinsically disordered regions calculation

Intrinsically disordered regions (IDRs) were generated by first computing residue-level disorder propensity scores with AIUPred [43, 44] and then applying a post-processing procedure following the MobiDB-lite protocol [45]. AIUPred scores were binarized using a disorder threshold of 0.5, yielding an initial residue-wise disorder state. To reduce spurious fragmentation of disordered segments, morphological smoothing was applied using consecutive dilation and erosion operations, followed by gap-merging to connect long disordered regions separated by short structured intervals. Long IDRs were then defined as contiguous disordered segments with a minimum length of 20 residues after post-processing. Proteins harboring at least one long IDR were classified as intrinsically disordered proteins (IDPs), whereas proteins lacking any long IDR were classified as non-IDPs. Likewise, PSPs with at least one long IDR were designated ID-PSPs, while PSPs without long IDRs were designated noID-PSPs.

### Positive dataset construction

Phase separating positive proteins were collected by integrating four curated LLPS-focused databases, including LLPSDB v2.0 [25], PhaSePro v1.1.0 [26], PhaSepDB v3.0 [27], and DrLLPS [28]. CD-CODE [46] was not used as a primary source to compile phase-separating proteins because it is a condensate-centric, community-editable “living” knowledgebase in which entries are continuously updated by contributors and maintainers, and its protein annotations primarily reflect condensate association rather than an intrinsic or autonomous phase-separation capability. Proteins shorter than 50 amino acids or longer than 3,000 amino acids were excluded. Proteins containing uncommon amino acids such as “BJOUXZ” were also excluded.

The combined positive records were subjected to systematic screening and deduplication. For LLPSDB, proteins from “Ambiguous” subset were excluded. For DrLLPS, only proteins with condensate information and tissue/cell annotations were included, and only those of the “Scaffold” or “Client” type were considered. Proteins from type “Client” reported in publications covering more than 10 entries from DrLLPS were excluded to avoid possible high-throughput annotations. Redundant records across databases were then collapsed to a non-redundant protein set, and the resulting taxonomic composition of the set was quantified. Protists were excluded from the set due to insufficient number of high-confidence phase separating positive proteins. A final set of 2,216 high-confidence PSPs was obtained and used as the positive benchmark set (Fig. 2; Additional file 1: Fig. S2A).

### Negative dataset construction

Candidate negative proteins were derived from the Swiss-Prot database with sequence length from 50 to 3,000 amino acids. To minimize contamination by proteins with reported condensate association, candidates showing sequence similarity to any protein included in the LLPS-focused databases used for positive set construction, as well as CD-CODE v1 [46], were removed based on BLASTP alignments using thresholds of sequence identity ≥ 40% and alignment coverage ≥ 70%. CD-CODE was used as an additional exclusion resource to reduce the risk that proteins with any reported condensate association were retained as negatives, thereby enforcing a stringent definition of the negative benchmark set. To reduce potential contamination by proteins used as positives in existing predictors, we removed the available training positive sets of all evaluated predictors from the benchmark negative set. This exclusion could not be applied to Droppler because its training sets were unavailable. In addition, to minimize the likelihood of retaining proteins with potential LLPS-related functional association, we further removed proteins annotated in BioGRID v4.4 [47] as first-degree interactors of the positive proteins. Candidates containing uncommon amino acids such as “BJOUXZ” were further excluded. The remaining proteins were classified into taxonomic groups. To reduce sequence redundancy while preserving taxon-specific diversity, CD-HIT clustering was performed independently within each taxonomic group using a sequence identity cutoff of 0.3.

Final negative set construction was performed by stratified sampling, with target counts guided by the taxonomic group and IDP/non-IDP composition of the positive set. Within each stratum, specific proteins were selected using an embedding-based diversity sampling strategy. Mean-pooled ESM-2 embeddings [48] were used to represent protein sequences, and a cosine-distance-based k-center greedy algorithm was applied to select proteins that maximally covered the sequence embedding space. This procedure ensured that the negative dataset spanned a broad and diverse sequence space while maintaining matched taxonomic group and disorder profiles relative to the positive set. The final negative benchmark set consisted of 12,071 proteins (Fig. 2; Additional file 1: Fig. S2B; Additional file 2: Table S1).

### Jaccard-index quantification of training-benchmark overlap

To quantify overlap between predictors’ training sets and benchmark datasets, we computed the Jaccard index, defined as $J=\mid A\cap B\mid /\mid A\cup B\mid$, where A and B denote the corresponding protein sets. For the Pintado-Grima benchmark, Jaccard indices were computed between each predictor’s training set and the Pintado-Grima dataset, with positive and negative sets analyzed separately where available. For our benchmark, because available training positive sets were removed before evaluation, training-benchmark overlap was assessed using each predictor’s available negative training set and the benchmark negative set.

### Sequence-similarity analysis within and between taxonomic groups

To quantify sequence similarity within and between taxonomic groups, we computed pairwise global sequence identity for all protein pairs separately for the positive and negative benchmark datasets. To assess overall sequence similarity stringently and avoid overestimation from short conserved motifs or domains, we used a global-alignment-based identity measure, rather than local alignment-based identity. Specifically, we computed pairwise global sequence identity for all protein pairs using the Parasail library [49], which implements the Needleman-Wunsch algorithm. For each protein pair, sequence identity was calculated as the number of identical aligned residues divided by the full alignment length. Pairwise identity values were grouped according to the taxonomic annotations of the two proteins and summarized as taxon-pair boxplots. Within-taxon comparisons refer to protein pairs from the same taxonomic group, whereas between-taxon comparisons refer to protein pairs from different taxonomic groups.

### Taxon-stratified characterization of LLPS-associated features

The LLPS-associated features were calculated for proteins in the constructed positive set. Firstly, amino-acid composition profiles were computed separately for each taxonomic group by calculating the mean residue frequency of each amino acid across proteins in that group. To highlight taxon-specific deviations, relative amino-acid composition ratio were computed with the following formula:

$$R_{s,aa}=\frac{fraction_{s,aa}}{\overline{fraction}_{aa}},$$

where ${fraction}_{s,aa}$ represents the mean residue frequency of amino acid (aa) in taxon s, ${\overline{fraction} }_{aa}$ means the average aa frequency of all protein samples.

In addition to amino-acid composition, fractions of the following amino acid groups were calculated: Aliphatic (A, V, L, I, M), Aromatic (F, W, Y), Polar uncharged (S, T, N, Q, C), Positively charged (K, R, H), Negatively charged (D, E), Hydrophilic (S, T, H, N, Q, E, D, K, R), Hydrophobic (V, I, L, F, W, Y, M), and Alpha Helix (V, I, Y, F, W, L). The fractions were defined as residue counts normalized by protein length.

A panel of disorder- and charge-related features were further calculated. Intrinsically disordered region (IDR) proportion was defined as the total length of all IDRs divided by protein length. The IDR binding score was defined as the proportion of residues whose ANCHOR [38] score exceeded 0.5 within IDRs, normalized by the full sequence length. IDR hydropathy was computed using the normalized Kyte-Doolittle hydrophobicity scale [50] implemented in localCIDER [37]. Net charge per residue (NCPR) was defined as the sequence-length-normalized net charge, computed by subtracting the counts of negatively charged residues (D and E) from positively charged residues (K, R, and H). The IDR Kappa (κ; charge patterning) was defined as the maximum κ value computed by localCIDER [37] across all IDRs identified in each protein.

Additionally, low-complexity score was calculated following the protocol used by PSAP [12]. Briefly, for each residue, a sliding window of 20 amino acids was constructed with the residue positioned at the window center. The number of unique amino-acid types within the window was then counted, and the central residue was assigned a low-complexity label of 1 when the unique count was ≤ 7, and 0 otherwise. For each protein, the low-complexity score was defined as the total number of low-complexity-labeled residues normalized by protein length.

To distinguish LLPS-associated signatures from taxon-specific proteome baselines, PSPs were compared against taxon-matched background proteins used for CD-HIT clustering, comprising 428,695 proteins in total (Additional file 1: Fig. S2B). Feature separation within each taxonomic group was quantified using Cliff’s δ, a rank-based effect size: δ > 0 indicates that PSPs tend to take higher values than the background, whereas δ < 0 indicates a shift toward lower values. Statistical significance was assessed using two-sided Mann–Whitney U tests with Benjamini–Hochberg false discovery rate (BH-FDR) correction.

### Homolog-restricted sensitivity analyses

To assess whether cross-taxon feature differences could be attributed to non-homologous comparisons, we performed a homolog-restricted sensitivity analysis using OMA orthogroup annotations. PSPs were first mapped to OMA orthogroups, and for each orthogroup we recorded the taxonomic groups represented by its PSP members. We initially sought orthogroups containing PSPs from all analyzed taxonomic groups, which would allow fully matched cross-taxon comparisons. When such broadly shared PSP orthogroups were unavailable, we further examined lower-order shared orthogroups. To avoid ambiguity from many-to-many homologous relationships, matched analyses were restricted to single-copy PSP orthogroups, in which each taxonomic group within the comparison contributed only one PSP. Only taxon sets with more than 10 shared PSP orthogroups were retained for feature-level statistical analysis. For retained two-taxon matched comparisons, feature values were compared across shared PSP orthogroups using two-sided paired Wilcoxon tests. *P*-values were adjusted using BH-FDR across the tested features and comparisons. Matched-pairs rank-biserial correlation was calculated as a paired effect-size measure. Positive and negative values indicate opposite directions of feature shift between the two taxa being compared.

### Taxonomy-aware benchmarking of PSP predictors

PSP predictors available before January 2026 were collected for benchmarking. A total of nineteen predictors including PLAAC [5], PScore [7], PSPer [8], FuzDrop [9], Droppler [10], DeePhase [11], PSAP [12], PhaSePred [13], PSPredictor [14], PICNIC [15], PSPire [16], FLFB [17], Seq2Phase [18], PSPHunter [19], PSTP [20], PhaseNet [21], MambaPhase [22], PSPsPredict [23], and ROBOT [24] were evaluated. The two PhaSePred variants (SaPS for self-assembling proteins and PdPS for partner-dependent proteins) and the two Seq2Phase variants (Seq2Phase-C targeting client proteins and Seq2Phase-S targeting scaffold proteins) were evaluated separately. For ROBOT, which supports both sequence-based and structure-based prediction modes, we evaluated the two input modes separately. The sequence-based version is denoted as ROBOT(seq), whereas the structure-based version is denoted as ROBOT(struct). Since R + Y [42] is a rule-based method that predicts saturation concentration (csat) for FUS-family-like disordered proteins from the abundance of arginine (R) and tyrosine (Y) residues, it was excluded from evaluation. Since the CatGRANULE [6] website no longer provides a prediction service, it was excluded from the evaluation. For predictors providing executable models or programmatic interfaces, predictions were generated according to published instructions. For predictors available only via web servers, prediction results were retrieved using automated Selenium-based scripts. For predictors providing only precomputed proteome-wide predictions without an executable pipeline, the published results were used directly. For PSTP, we used the mixed-dataset-trained model and took its sequence-level prediction score as the final phase-separation propensity for each protein. For PSPire, we used the version including the phosphorylation frequency feature for human proteins and the version excluding this feature for non-human species.

Benchmark evaluation was performed on the six taxonomic groups of the benchmark dataset: Animals(M), Animals(NM), Fungi, Plants, Prokaryotes, and Viruses. The two PhaSePred variants (SaPS and PdPS) were excluded from evaluation on the Prokaryotes subset due to limited protein support; while PhaSePred, PSPire, ROBOT(struct), and PICNIC were excluded from evaluation on the Viruses subset for the same reason. In addition, evaluation was performed on a pooled benchmark subset combining Animals(M), Animals(NM), Fungi, and Plants. Prokaryotes and Viruses subsets were excluded from pooled analyses due to limited predictor support.

To ensure fair comparison, proteins not supported by any predictor were excluded from the constructed evaluation sets. For the main benchmark analysis, we mitigated potential training-data leakage by removing proteins appearing in the available training positive sets of all evaluated predictors from the evaluation sets. Because no response was received from the Droppler authors, no explicit training-positive exclusion could be applied for Droppler. Results from this main evaluation are presented in the main figures.

To further assess whether predictor performance was influenced by sequence similarity between predictor training positives and benchmark positives, we performed an additional training-similarity filtering analysis. In this analysis, after removing the available training positives of evaluated predictors, we used BLASTP to compare the remaining benchmark positives against the union of available predictor training positive sets. Within each taxonomic group, benchmark positives showing > 30% sequence identity to any available training-positive sequence were removed. Predictor evaluation was then repeated on the remaining taxonomic groups with sufficient positive proteins.

For both the main benchmark analysis and the additional training-similarity filtering analysis, predictor evaluation was performed using balanced positive and negative sets within each taxonomic group. Specifically, negatives were randomly sampled to match the number of positives, yielding a 1:1 negative-to-positive ratio. This procedure was repeated ten times using different random seeds. To improve the utilization of available negatives, negative sampling was performed without replacement across repeats whenever possible; when the remaining negatives within a given sampling stratum were insufficient, sampling was restarted from the full negative pool of that stratum. In each repeat, the sampled negatives were additionally constrained to match the IDP/non-IDP composition of the positive set within the same taxonomic group. Predictor performance metrics were calculated for each repeat and summarized as mean and variance across the ten balanced subsampled datasets.

Predictor performance across taxonomic groups was quantified using both threshold-free metrics (AUROC, AUPRC, and AUPRC ratio) and threshold-dependent metrics (MCC, F1-score, precision, recall, specificity, accuracy, and positive likelihood ratio). The AUPRC ratio was defined as the observed AUPRC divided by the random expectation, where the random expectation equals the positive fraction in the evaluated set, while the positive likelihood ratio (LR +) was defined as sensitivity/(1 − specificity). Calculation of threshold-dependent metrics requires binarization of continuous predictor outputs. When explicit decision thresholds were defined by the original authors, those thresholds were applied: 4 for PScore, and 0.6 for FuzDrop. For PLAAC, proteins with a COREscore (COREscore not NaN) were classified as positive. For FLFB, proteins assigned the output label “LLPS” were classified as positive. For the remaining predictors (PSPer, Droppler, DeePhase, PSAP, PhaSePred, PSPredictor, PICNIC, PSPire, Seq2Phase, PSPHunter, PSTP, PhaseNet, MambaPhase, PSPsPredict, and ROBOT), for which explicit thresholds were not provided, the decision threshold that maximized MCC on the corresponding evaluation set was used. The threshold yielding the maximum MCC was obtained by scanning thresholds from 0.01 to 0.99 in steps of 0.01. Thresholds were determined independently for each predictor across ten independently generated negative-set subsamples.

Additionally, within each taxonomic group, positives were further stratified by disorder status into ID-PSPs and noID-PSPs to quantify performance differences between these two classes. Performance on ID-PSPs and noID-PSPs was evaluated only in the Animals(M) subset, because the other subsets contained too few noID-PSPs (fewer than 30) to support reliable evaluation.

## Supplementary Information

Additional file 1: Supplementary Figures: Figs. S1-S11.

Additional file 2: Table S1. List of proteins in benchmark positive and negative sets.

Additional file 3: Table S2. List of evaluated phase-separating protein predictors.

Additional file 4: Table S3. Predictor performance across six taxonomic groups in the benchmark dataset.

Additional file 5: Table S4. Predictor performance on the Animals, Fungi, and Plants subsets after training-positive removal and training-similarity filtering.

## Data Availability

The following public databases were used for the construction of benchmark datasets: LLPSDB v2.0 [25], PhaSePro v1.1.0 [26], PhaSepDB v3.0 [27], DrLLPS [28], CD-CODE v1 [46], BioGRID v4.4 [47], and UniProt [51]. Datasets and coding scripts are available at https://github.com/TongjiZhanglab/taxonomy-aware-benchmark [52] and released to Zenodo 10.5281/zenodo.20077494 [53] under a MIT license.

## References

[1] Alberti S, Gladfelter A, Mittag T. Considerations and challenges in studying liquid-liquid phase separation and biomolecular condensates. Cell. 2019;176:419–34. 30682370 10.1016/j.cell.2018.12.035 PMC6445271

[2] Lyon AS, Peeples WB, Rosen MK. A framework for understanding the functions of biomolecular condensates across scales. Nat Rev Mol Cell Biol. 2021;22:215–35. 33169001 10.1038/s41580-020-00303-z PMC8574987

[3] Alberti S, Dormann D. Liquid-liquid phase separation in disease. Annu Rev Genet. 2019;53:171–94. 31430179 10.1146/annurev-genet-112618-043527

[4] Alberti S, Hyman AA. Biomolecular condensates at the nexus of cellular stress, protein aggregation disease and ageing. Nat Rev Mol Cell Biol. 2021;22:196–213. 33510441 10.1038/s41580-020-00326-6

[5] Lancaster AK, Nutter-Upham A, Lindquist S, King OD. PLAAC: a web and command-line application to identify proteins with prion-like amino acid composition. Bioinformatics. 2014;30:2501–2. 24825614 10.1093/bioinformatics/btu310 PMC4147883

[6] Bolognesi B, Gotor NL, Dhar R, Cirillo D, Baldrighi M, Tartaglia GG, et al. A concentration-dependent liquid phase separation can cause toxicity upon increased protein expression. Cell Rep. 2016;16:222–31. 27320918 10.1016/j.celrep.2016.05.076 PMC4929146

[7] Vernon RM, Chong PA, Tsang B, Kim TH, Bah A, Farber P, et al. Pi-Pi contacts are an overlooked protein feature relevant to phase separation. Elife. 2018;7:e31486. 29424691 10.7554/eLife.31486 PMC5847340

[8] Orlando G, Raimondi D, Tabaro F, Codicè F, Moreau Y, Vranken WF. Computational identification of prion-like RNA-binding proteins that form liquid phase-separated condensates. Bioinformatics. 2019;35:4617–23. 30994888 10.1093/bioinformatics/btz274

[9] Hardenberg M, Horvath A, Ambrus V, Fuxreiter M, Vendruscolo M. Widespread occurrence of the droplet state of proteins in the human proteome. Proc Natl Acad Sci U S A. 2020;117:33254–62. 33318217 10.1073/pnas.2007670117 PMC7777240

[10] Raimondi D, Orlando G, Michiels E, Pakravan D, Bratek-Skicki A, Van Den Bosch L, et al. In silico prediction of in vitro protein liquid-liquid phase separation experiments outcomes with multi-head neural attention. Bioinformatics. 2021;37:3473–9. 33983381 10.1093/bioinformatics/btab350

[11] Saar KL, Morgunov AS, Qi R, Arter WE, Krainer G, Lee AA, et al. Learning the molecular grammar of protein condensates from sequence determinants and embeddings. Proc Natl Acad Sci U S A. 2021;118:e2019053118. 33827920 10.1073/pnas.2019053118 PMC8053968

[12] van Mierlo G, Jansen JR, Wang J, Poser I, van Heeringen SJ, Vermeulen M. Predicting protein condensate formation using machine learning. Cell Rep. 2021;34:108705. 33535034 10.1016/j.celrep.2021.108705

[13] Chen Z, Hou C, Wang L, Yu C, Chen T, Shen B, et al. Screening membraneless organelle participants with machine-learning models that integrate multimodal features. Proc Natl Acad Sci U S A. 2022;119:e2115369119. 35687670 10.1073/pnas.2115369119 PMC9214545

[14] Chu X, Sun T, Li Q, Xu Y, Zhang Z, Lai L, et al. Prediction of liquid-liquid phase separating proteins using machine learning. BMC Bioinformatics. 2022;23:72. 35168563 10.1186/s12859-022-04599-w PMC8845408

[15] Hadarovich A, Singh HR, Ghosh S, Scheremetjew M, Rostam N, Hyman AA, et al. PICNIC accurately predicts condensate-forming proteins regardless of their structural disorder across organisms. Nat Commun. 2024;15:10668. 39663388 10.1038/s41467-024-55089-x PMC11634905

[16] Hou S, Hu J, Yu Z, Li D, Liu C, Zhang Y. Machine learning predictor PSPire screens for phase-separating proteins lacking intrinsically disordered regions. Nat Commun. 2024;15:2147. 38459060 10.1038/s41467-024-46445-y PMC10923898

[17] Liao S, Zhang Y, Han X, Wang T, Wang X, Yan Q, et al. A sequence-based model for identifying proteins undergoing liquid-liquid phase separation/forming fibril aggregates via machine learning. Protein Sci. 2024;33:e4927. 38380794 10.1002/pro.4927 PMC10880426

[18] Miyata K, Iwasaki W. Seq2Phase: language model-based accurate prediction of client proteins in liquid-liquid phase separation. Bioinform Adv. 2024;4:vbad189. 38205268 10.1093/bioadv/vbad189 PMC10777356

[19] Sun J, Qu J, Zhao C, Zhang X, Liu X, Wang J, et al. Precise prediction of phase-separation key residues by machine learning. Nat Commun. 2024;15:2662. 38531854 10.1038/s41467-024-46901-9 PMC10965946

[20] Feng M, Liu L, Xian Z-N, Wei X, Li K, Yan W, et al. PSTP: accurate residue-level phase separation prediction using protein conformational and language model embeddings. Brief Bioinform. 2025;26:bbaf171. 40315433 10.1093/bib/bbaf171 PMC12047702

[21] He X, Guan J, Xie P, Wang F, Zhao Z, Liu X, et al. PhaseNet: a computational framework for identifying phase-separating proteins based on protein language model. Int J Biol Macromol. 2025;334:149044. 41241351 10.1016/j.ijbiomac.2025.149044

[22] Huang J, Zhang Y, Ren S, Wang Z, Jin X, Lu X, et al. MambaPhase: deep learning for liquid–liquid phase separation protein classification. Brief Bioinform. 2025;26:bbaf230. 40421658 10.1093/bib/bbaf230 PMC12107247

[23] Li W, Deng X, Xiang C, Shen H, Zhang Y. Prediction of liquid–liquid phase separation proteins based on protein language model. Brief Bioinform. 2025;26:bbaf681. 41405960 10.1093/bib/bbaf681 PMC12710474

[24] Monti M, Fiorentino J, Miltiadis-Vrachnos D, Bini G, Cotrufo T, Sanchez de Groot N, et al. CatGRANULE 2.0: accurate predictions of liquid-liquid phase separating proteins at single amino acid resolution. Genome Biol. 2025;26:33. 39979996 10.1186/s13059-025-03497-7 PMC11843755

[25] Wang X, Zhou X, Yan Q, Liao S, Tang W, Xu P, et al. LLPSDB v2.0: an updated database of proteins undergoing liquid–liquid phase separation in vitro. Bioinformatics. 2022;38:2010–4. 35025997 10.1093/bioinformatics/btac026 PMC8963276

[26] Mészáros B, Erdős G, Szabó B, Schád É, Tantos Á, Abukhairan R, et al. PhaSePro: the database of proteins driving liquid–liquid phase separation. Nucleic Acids Res. 2020;48:D360–7. 31612960 10.1093/nar/gkz848 PMC7145634

[27] You KQ, Li RY, Lian RX, Li YX, Yang HZN, Zhou YR, et al. PhaSepDB 3.0: a comprehensive knowledgebase of phase separation-related proteins from AI-assisted curation. Nucleic Acids Res. 2026;54:D445–50. 41062450 10.1093/nar/gkaf973 PMC12807760

[28] Ning W, Guo Y, Lin S, Mei B, Wu Y, Jiang P, et al. DrLLPS: a data resource of liquid–liquid phase separation in eukaryotes. Nucleic Acids Res. 2020;48:D288–95. 31691822 10.1093/nar/gkz1027 PMC7145660

[29] Pintado-Grima C, Barcenas O, Arribas-Ruiz E, Iglesias V, Burdukiewicz M, Ventura S. Comprehensive protein datasets and benchmarking for liquid-liquid phase separation studies. Genome Biol. 2025;26:198. 40629388 10.1186/s13059-025-03668-6 PMC12239342

[30] King OD, Gitler AD, Shorter J. The tip of the iceberg: RNA-binding proteins with prion-like domains in neurodegenerative disease. Brain Res. 2012;1462:61–80. 22445064 10.1016/j.brainres.2012.01.016 PMC3372647

[31] Banani SF, Lee HO, Hyman AA, Rosen MK. Biomolecular condensates: organizers of cellular biochemistry. Nat Rev Mol Cell Biol. 2017;18:285–98. 28225081 10.1038/nrm.2017.7 PMC7434221

[32] Elnaggar A, Heinzinger M, Dallago C, Rehawi G, Wang Y, Jones L, et al. ProtTrans: toward understanding the language of life through self-supervised learning. IEEE Trans Pattern Anal Mach Intell. 2021;44:7112–27. 10.1109/TPAMI.2021.3095381 34232869

[33] McInnes L, Healy J, Melville J. UMAP: uniform manifold approximation and projection for dimension reduction. 2018. arXiv preprint arXiv:180203426.

[34] Jiang S, Li H, Zhang L, Mu W, Zhang Y, Chen T, et al. Generic Diagramming Platform (GDP): a comprehensive database of high-quality biomedical graphics. Nucleic Acids Res. 2025;53:D1670–6. 39470721 10.1093/nar/gkae973 PMC11701665

[35] Lex A, Gehlenborg N, Strobelt H, Vuillemot R, Pfister H. UpSet: visualization of intersecting sets. IEEE Trans Vis Comput Graph. 2014;20:1983–92. 26356912 10.1109/TVCG.2014.2346248 PMC4720993

[36] Fu L, Niu B, Zhu Z, Wu S, Li W. CD-HIT: accelerated for clustering the next-generation sequencing data. Bioinformatics. 2012;28:3150–2. 23060610 10.1093/bioinformatics/bts565 PMC3516142

[37] Holehouse AS, Das RK, Ahad JN, Richardson MO, Pappu RV. CIDER: resources to analyze sequence-ensemble relationships of intrinsically disordered proteins. Biophys J. 2017;112:16–21. 28076807 10.1016/j.bpj.2016.11.3200 PMC5232785

[38] Dosztányi Z, Mészáros B, Simon I. ANCHOR: web server for predicting protein binding regions in disordered proteins. Bioinformatics. 2009;25:2745–6. 19717576 10.1093/bioinformatics/btp518 PMC2759549

[39] Erdős G, Pajkos M, Dosztányi Z. IUPred3: prediction of protein disorder enhanced with unambiguous experimental annotation and visualization of evolutionary conservation. Nucleic Acids Res. 2021;49:W297–303. 34048569 10.1093/nar/gkab408 PMC8262696

[40] Cliff N. Dominance statistics - ordinal analyses to answer ordinal questions. Psychol Bull. 1993;114:494–509.

[41] Altschul SF, Gish W, Miller W, Myers EW, Lipman DJ. Basic local alignment search tool. J Mol Biol. 1990;215:403–10. 2231712 10.1016/S0022-2836(05)80360-2

[42] Schwartz JC, Cech TR, Parker RR. Biochemical properties and biological functions of FET proteins. Annu Rev Biochem. 2015;84:355–79. 25494299 10.1146/annurev-biochem-060614-034325 PMC9188303

[43] Erdős G, Dosztányi Z. AIUPred: combining energy estimation with deep learning for the enhanced prediction of protein disorder. Nucleic Acids Res. 2024;52:W176–81. 38747347 10.1093/nar/gkae385 PMC11223784

[44] Erdős G, Deutsch N, Dosztányi Z. AIUPred–binding: energy embedding to identify disordered binding regions. J Mol Biol. 2025;437:169071. 40133781 10.1016/j.jmb.2025.169071

[45] Mehdiabadi M, Blum M, Tesei G, von Bülow S, Lindorff-Larsen K, Tosatto SC, et al. MobiDB-lite 4.0: faster prediction of intrinsic protein disorder and structural compactness. Bioinformatics. 2025;41:btaf297. 10.1093/bioinformatics/btaf297 PMC12122076 40347452

[46] Rostam N, Ghosh S, Chow CFW, Hadarovich A, Landerer C, Ghosh R, et al. CD-CODE: crowdsourcing condensate database and encyclopedia. Nat Methods. 2023;20:673–6. 37024650 10.1038/s41592-023-01831-0 PMC10172118

[47] Oughtred R, Rust J, Chang C, Breitkreutz BJ, Stark C, Willems A, et al. The BioGRID database: a comprehensive biomedical resource of curated protein, genetic, and chemical interactions. Protein Sci. 2021;30:187–200. 33070389 10.1002/pro.3978 PMC7737760

[48] Lin Z, Akin H, Rao R, Hie B, Zhu Z, Lu W, et al. Evolutionary-scale prediction of atomic-level protein structure with a language model. Science. 2023;379:1123–30. 36927031 10.1126/science.ade2574

[49] Daily J. Parasail: SIMD C library for global, semi-global, and local pairwise sequence alignments. BMC Bioinformatics. 2016;17:81. 26864881 10.1186/s12859-016-0930-z PMC4748600

[50] Kyte J, Doolittle RF. A simple method for displaying the hydropathic character of a protein. J Mol Biol. 1982;157:105–32. 7108955 10.1016/0022-2836(82)90515-0

[51] Consortium TU. UniProt: the Universal Protein Knowledgebase in 2025. Nucleic Acids Res. 2025;53:D609–17. 39552041 10.1093/nar/gkae1010 PMC11701636

[52] Hou S, Shen H, Zhang Y. Taxonomy-aware, disorder-matched benchmarking of phase-separating protein predictors. GitHub. 2026. https://github.com/TongjiZhanglab/taxonomy-aware-benchmark. 10.1186/s13059-026-04164-1 PMC13508433 42310761

[53] Hou S, Shen H, Zhang Y. Taxonomy-aware, disorder-matched benchmarking of phase-separating protein predictors. Zenodo. 2026. 10.5281/zenodo.20077494. 10.1186/s13059-026-04164-1 PMC13508433 42310761

