# Supplementary material for: Taxonomy-aware, disorder-matched benchmarking of phase-separating protein predictors
Source: Genome Biol. 2026 Jun 17;27:273. doi: 10.1186/s13059-026-04164-1 (PMC13508433; doi:10.1186/s13059-026-04164-1)
Supplement: Supplementary file 1 [file 13059_2026_4164_MOESM1_ESM.docx]

**Taxonomy-aware, disorder-matched benchmarking of phase-separating protein predictors**

Shuang Hou^1,#^, Hexin Shen^1,#^, Yong Zhang^1,*^

^1^State Key Laboratory of Cardiovascular Diseases and Medical Innovation Center, Institute for Regenerative Medicine, Department of Neurosurgery, Shanghai East Hospital, Shanghai Key Laboratory of Signaling and Disease Research, Frontier Science Center for Stem Cell Research, School of Life Sciences and Technology, Tongji University, Shanghai, 200092, China

^#^These authors contributed equally

^*^Correspondence: [yzhang@tongji.edu.cn](mailto:yzhang@tongji.edu.cn)

**SUPPLEMENTARY FIGURES**

**Fig S1.** Training-set size, intrinsically disordered regions (IDRs) content, and proportion of proteins with IDRs (IDPs) vary widely across PSP predictors and the Pintado-Grima benchmark. Predictors are grouped by the taxonomic scope of their training data (as indicated below): multi-taxon predictors (trained on multiple taxa) and single-taxon predictors (trained on a single taxon). **A** Sample sizes of the positive and negative training sets for PSP predictors and the Pintado-Grima benchmark. Bar heights indicate sample counts (log-scaled y axis), with counts annotated above bars. **B** Distributions of IDR proportion for proteins in the positive and negative training sets for PSP predictors and the Pintado-Grima benchmark. Violin plots show the full distribution with an overlaid boxplot. The central line indicates the median. The box represents the interquartile range (IQR), covering the 25th to 75th percentile. Whiskers extend to the data’s minima and maxima within 1.5 × IQR. “NA” indicates that the corresponding set was not available for analysis. **C** Proportions of proteins classified as IDPs and non-IDPs in the positive and negative training sets for PSP predictors and the Pintado-Grima benchmark. Stacked bars show the fraction of proteins classified as IDP versus non-IDP within each set. In this study, an IDP is defined as a protein containing at least one long IDR, where a long IDR is a contiguous disordered segment of at least 20 amino acids after AIUPred-based disorder prediction and MobiDB-lite-style post-processing. A non-IDP is defined as a protein without such a long IDR. **D** Jaccard index between the training sets of PSP predictors and the Pintado-Grima benchmark dataset, calculated separately for positive and negative sets. “NA” indicates that the corresponding set was not available for analysis.

**Fig S2.** Overview of taxonomy-aware benchmark dataset construction. Sankey diagram summarizing the workflow used to derive high-confidence positives and a taxon-matched negative pool. **A** For the positive dataset, records from curated LLPS-focused resources (DrLLPS, PhaSepDB, PhaSePro and LLPSDB) were merged, screened and deduplicated to obtain the final positive set (n = 2,216). **B** For the negative dataset, Swiss-Prot proteins were filtered to obtain a pool of likely non-PSPs. Specifically, proteins with known or putative LLPS association were excluded, including the compiled PSPs, proteins showing sequence similarity to PSPs, known interactors of positive proteins, and proteins used as positives in the available training sets of evaluated predictors. Redundancy reduction was then performed within each taxonomic group (Animals(M), Animals(NM), Fungi, Plants, Prokaryotes and Viruses) to enable taxonomy-aware negative selection. Stratified sampling guided by the positive set’s taxonomic-group and IDP/non-IDP composition generated the final negative set (n = 12,071). Numbers indicate protein counts at each step.

**Fig S3.** Overview of taxonomy-aware benchmark dataset composition. **A** UpSet plot showing overlap among PSP records collected from four curated LLPS-focused resources (PhaSepDB, PhaSePro, LLPSDB and DrLLPS). Bars indicate the size of each intersection (top) and the total contribution of each resource (left). Filled dots denote the combination of resources defining each intersection; percentages report the fraction of the final positive set represented by each intersection. **B** Taxonomic-group composition of the positive and negative benchmark sets. Stacked bars show protein counts per taxonomic group (Animals(M), Animals(NM), Fungi, Plants, Prokaryotes and Viruses), partitioned into IDP and non-IDP subsets; numbers above bars indicate total counts per group. **C** Taxonomic-group distributions of the positive and negative benchmark sets. Pie charts show the proportions of proteins assigned to each major taxonomic group in the positive set (left) and negative set (right), including Animals(M), Animals(NM), Fungi, Plants, Prokaryotes, and Viruses. The negative set was sampled to match the taxonomic composition of the positive set; accordingly, the two sets show closely matched taxonomic-group distribution. **D** Protein length distributions of the positive benchmark set, negative benchmark set, and proteome background.

**Fig S4.** Sequence similarity within and between taxonomic groups. Pairwise global sequence identity was computed for all protein pairs in the positive and negative benchmark datasets using Needleman-Wunsch global alignment implemented in Parasail. Sequence identity was defined as the fraction of identical residues over the full alignment length. **A** Pairwise global identity heatmaps for the positive and negative datasets. Each entry represents the global sequence identity between two proteins, with proteins grouped by taxonomic annotation. **B** Taxon-pair boxplots for the positive and negative datasets, summarizing the distributions of pairwise global identity values for each within- or between-taxon comparison. Boxplots show the median, interquartile range, and whiskers extending to the most extreme values within 1.5 × IQR.

**Fig S5.** Distributions of representative sequence and biophysical features for positive proteins across taxa, including IDR binding score, IDR hydropathy, net charge per residue, and the fractions of residues belonging to hydrophilic (S, T, H, N, Q, E, D, K, R), hydrophobic (V, I, L, F, W, Y, M), aromatic (F, W, Y), aliphatic (A, V, L, I, M) and α-helix-promoting (V, I, Y, F, W, L) groups. Box plots show the median (central line), interquartile range (IQR; 25th-75th percentiles), and whiskers extending to the most extreme values within 1.5 × IQR. Letters denote statistical groupings from pairwise comparisons among taxonomic groups within each feature. For each feature, two-sided Mann-Whitney U tests were performed between taxonomic groups, followed by BH-FDR correction. Taxonomic groups assigned the same letter are not significantly different, whereas taxonomic groups assigned different letters differ significantly at BH-FDR < 0.05.

**Fig S6.** Homolog-restricted paired comparison of PSP features for Animals(M)-Fungi and Animals(M)-Animals(NM). Heatmap colors indicate matched-pairs rank-biserial correlation. Asterisks indicate statistical significance from two-sided paired Wilcoxon tests after BH-FDR correction.

**Fig S7.** Taxon-specific functional and domain enrichments of phase-separating proteins. Enrichment of Pfam domains (A) and Gene Ontology (GO) biological process terms (B) in positives across six taxonomic groups (Animals(M), Animals(NM), Fungi, Plants, Prokaryotes and Viruses). For each taxon, enrichment was calculated by comparing positives against the corresponding taxon-matched proteome background. Dot color encodes log2 fold enrichment (color bar), and dot size reflects enrichment significance (BH-FDR). Grey markers denote non-significant terms.

**Fig S8.** Distribution of predictor performance across ten balanced negative-sampling repeats on the Animals(M) subset. For each repeat, negatives were randomly sampled to match the number of positives after removal of available predictor training positives, yielding balanced 1:1 evaluation set. Box plots show AUROC (A) and AUPRC (B) values across the ten repeats for each predictor, with the median indicated by the central line, the interquartile range (IQR; 25th-75th percentiles) by the box, and whiskers extending to the most extreme values within 1.5 × IQR.

**Fig S9.** Predictor performance after training-positive removal and 30% training-similarity filtering. **A** Performance of predictors on the benchmark dataset after removing available training positive sets of all evaluated predictors, except for Droppler, whose training positives were unavailable. Predictor performance is shown for the pooled dataset combining Animals(M), Animals(NM), Fungi, and Plants (green squares); Prokaryotes and Viruses were excluded because only a subset of predictors supported these taxa, enabling a consistent comparison across methods. **B-D** Predictor performance after additional 30% training-similarity filtering. Benchmark positives were first filtered to remove available predictor training positives and then further filtered within each taxonomic group to exclude proteins sharing >30% sequence identity with any available predictor training positive. Prokaryotes, Animals(NM), and Viruses retained fewer than 30 positives after filtering and were therefore excluded. Predictor performance is shown for Animals(M), Fungi, and Plants.

**Fig S10.** Predictor performance on phase-separating proteins with IDRs (ID-PSPs) and phase-separating proteins without IDRs (noID-PSPs) on the Animals(M) subset. Other subsets were not analyzed in this stratified manner because they contained too few noID-PSPs to support reliable evaluation. For each predictor, mean AUROC values across ten negative subsampling runs are shown separately for ID-PSPs (blue) and noID-PSPs (orange). Vertical connectors indicate the within-predictor performance gap between the two subsets.

**Fig S11.** Overlap between the negative training sets of PSP predictors and the negative set of benchmark dataset quantified by the Jaccard index. Predictors are grouped by the taxonomic scope of their training data (as indicated below): multi-taxon predictors (trained on multiple taxa) and single-taxon predictors (trained on a single taxon). Available training positive sets of predictors were removed from the evaluation sets before benchmarking; therefore, overlap was only assessed on the negative sets. Since the three rule-based, unsupervised models (PLAAC, PScore, and PSPer) do not require training data, they were not shown. “NA” indicates that the corresponding set was not available for analysis.
